# Athletic clothing style and comfort: Associations with appearance monitoring, social physique anxiety, and task concentration among women

**DOI:** 10.1177/17455057261443139

**Published:** 2026-04-18

**Authors:** Madison F. Vani, Catherine M. Sabiston, Elizabeth Cox, April Karlinsky, Timothy N. Welsh

**Affiliations:** 1 Faculty of Kinesiology & Physical Education, University of Toronto, ON, Canada; 2 Division of Population Sciences, Department of Medical Oncology, Dana-Farber Cancer Institute, Boston, MA, USA; 3 Harvard Medical School, Boston, MA, USA; 4 Department of Kinesiology, College of Natural Sciences, California State University, San Bernardino, CA, USA

**Keywords:** body image, clothing comfort, objectification, young adults

## Abstract

**Background:**

Self-objectification increases women’s appearance monitoring and social physique anxiety (SPA) and diminishes their task-related concentration. Clothing style (tight and revealing (TR) versus loose and concealing (LC)) and preferences (comfort) may predict self-objectification-informed outcomes.

**Objectives:**

This study was designed to examine the influence of athletic clothing style on appearance monitoring, SPA, concentration, and clothing comfort, and tested the association between clothing comfort and self-objectification variables among women.

**Design:**

We used an experimental design conducted in a single laboratory session.

**Methods:**

Participants (*N* = 73; *M*_age_ = 20.77 ± 3.15 years) were randomly assigned to wear TR or LC athletic clothing and then completed SPA, concentration, and clothing comfort measures, and had their body awareness primed. Two raters independently counted appearance monitoring behaviours (e.g., mirror gazing, adjusting clothing) during task performance.

**Results:**

Women in the TR group engaged in more appearance monitoring and rated their clothing as more uncomfortable compared to the LC group. Poorer comfort ratings predicted higher SPA and lower concentration, after adjusting for trait self-objectification, clothing style, and body size perception.

**Conclusion:**

The present study generated methodological, theoretical, and applied impacts. First, this study introduced a novel behavioural method for assessing appearance monitoring through systematic video coding, offering a new approach for examining appearance monitoring in experimental contexts. Second, extending prior research, findings uniquely indicate clothing style is related to objective forms of appearance monitoring. Finally, results suggest encouraging women to wear comfortable exercise clothing may help reduce SPA and improve concentration, highlighting clothing comfort as an important individual difference and extending theoretical understanding of contextual factors relevant to Objectification Theory. Future research should examine objective and covert forms of appearance monitoring and further explore how variations in athletic clothing style and comfort relate to women’s body image and cognitive resources.

## Introduction

According to Objectification Theory,^1^ women in Western societies are commonly sexually objectified (i.e., a woman is regarded only as a body to be used by others), causing them to persistently view their bodies as objects (i.e., self-objectification). The tendency to self-objectify leads women to allocate cognitive resources towards their physical body, prompting increased appearance monitoring (i.e., preoccupation with appearance through behaviours such as adjusting clothing).^2^ Based on Objectification Theory, appearance monitoring brings about negative psychological outcomes including heightened social physique anxiety (SPA, i.e., the anxiety experienced regarding negative evaluations of the visible body by others)^3^ and diminished concentration on mental or physical tasks.^1,4^ Higher appearance monitoring and SPA and lower concentration can lead to worsened cognitive and motor performance and decreased well-being.^1,5–7^

Clothing (e.g., style and comfort) is one contextual factor that can elicit self-objectification and influence how women experience their bodies.^8^ Clothing style has often been used to experimentally manipulate state self-objectification.^8^ Tight and revealing (TR) clothing (i.e., form-fitting clothing that exposes the body or body shape such as a swimsuit) is commonly used to induce state self-objectification, while loose and concealing (LC) clothing (i.e., loose-fitting clothing that does not expose the body or body shape such as a sweater or other baggy clothing) is used as a non-objectifying comparison.^2,9^ Researchers have demonstrated that women wearing TR clothing compared to LC clothing perform worse on cognitive tasks, including a math test^2,10^ and a Stroop task assessing attentional processes.^9^ Women wearing TR athletic clothing (spandex tank top and shorts) also demonstrated worse motor performance on a visuomotor aiming task than their peers wearing LC clothing (basketball shorts and loose t-shirt).^11^ Increased state self-objectification associated with wearing TR clothing may contribute to greater appearance monitoring, which in turn leads to cognitive resources that are allocated towards one’s physical body, depleting available resources for the goal task.^2,9,11^

Although prior research has explored how clothing style influences cognitive and motor outcomes, less is known about the direct impact of athletic clothing style on appearance monitoring, SPA, and concentration – psychosocial and cognitive outcomes reflect the amount of cognitive resources dedicated to non-goal processes. Existing findings suggest women wearing TR clothing report greater appearance monitoring than those wearing LC clothing,^12^ and women wearing swimsuits report higher SPA compared to those wearing sweaters.^6^ When examining women’s athletic clothing, self-reported appearance monitoring was positively associated with tight athletic clothing, and negatively associated with loose athletic clothing.^13^ Women wearing TR athletic clothing also reported more appearance-related statements and greater general anxiety than those wearing LC athletic clothing.^14^ However, the influence of *athletic* clothing style on SPA and an *objective* measure of appearance monitoring (e.g., mirror checking, adjusting clothing) has yet to be examined.^8^ Likewise, the impact of athletic clothing style on concentration remains unexplored.^8^ One study demonstrated women wearing swimsuits reported lower intrinsic task motivation than women wearing sweaters.^6^ Since intrinsic motivation reflects participation in an activity for enjoyment or satisfaction, it is occasionally used as an indicator of flow experiences (i.e., full absorption in a task).^6,15^ As flow represents a state similar to concentration,^1,4^ these findings suggest that clothing style could also influence women’s concentration. Given that athletic clothing is commonly worn by women across exercise, sport, and leisure settings, understanding how clothing style impacts appearance monitoring, SPA, and concentration has meaningful implications not only for performance but also for women’s engagement and confidence in these settings. The style of athletic clothing chosen may inadvertently heighten self-objectification processes, increasing appearance monitoring and SPA, and reducing concentration, factors that could negatively impact performance and elevate injury risk. Accordingly, the present study’s first objective was to examine whether scores on appearance monitoring, SPA, and concentration differed based on the style of athletic clothing worn by women (TR versus LC).

In addition to the style of clothing, understanding women’s clothing preferences is important. One’s preference for clothing is often predicated on how comfortable or uncomfortable the TR or LC clothing is to that individual. TR clothing can be perceived to be uncomfortable and objectifying for some women,^12,16^ whereas other women may find TR clothing comfortable and enjoyable to wear.^17^ The complex relationship between style of clothing and perceptions of comfort persists in the context of activewear. Some women have reported that tight athletic clothing is not comfortable and limits their engagement in physical activities,^16,17^ whereas other women have reported that LC athletic clothing may get in the way of properly performing certain movements (e.g., kick in kickboxing) and can cause greater discomfort in activity.^17^ Thus, a second objective of the current study was to test whether scores on clothing comfort differed based on the athletic clothing style worn by women (TR versus LC).

It is reasonable to predict that clothing comfort may be associated with women’s appearance monitoring, SPA, and concentration. Notably, clothing comfort ratings have been related to the allocation of cognitive resources within non-athletic domains.^18,19^ For example, participants who wore wool clothing reported lower comfort ratings and performed worse on a visual vigilance task (used to assess cognitive performance) compared to those who wore cotton.^18^ Relatedly, students who rated their self-selected clothing as more comfortable achieved higher math exam scores.^19^ These findings suggest that comfort can influence cognitive performance. In terms of athletic clothing, qualitative research indicates a similar pattern. Women have reported that when their athletic clothing feels comfortable, their focus is not on their appearance, but rather their activity,^17^ conceivably reducing appearance monitoring and SPA while enhancing concentration. In fact, women have described that wearing comfortable clothing (regardless of clothing style) facilitates their ability to be immersed in their activity.^17^ Together, these findings highlight the potential psychological importance of perceived comfort, yet experimental research explicitly testing these relationships with athletic clothing is lacking. Understanding if clothing comfort relates to appearance monitoring, SPA, and concentration is crucial, because discomfort may elicit self-objectification and associated consequences. Therefore, the third objective of the present study was to examine whether ratings of clothing comfort were associated with scores on appearance monitoring, SPA, and concentration among women, providing a novel test of these relationships in an experimental setting.

In summary, the purpose of the present study was to examine the influence of athletic clothing style (i.e., TR versus LC) on appearance monitoring, SPA, concentration, and ratings of clothing comfort among women, and test the association among these variables. Participants, who were part of a larger study,^11^ were randomly assigned to wear TR or LC clothing and completed a series of tasks in a room with a mirror. Appearance monitoring was defined as behaviours in which the participant overtly attended to their observable body, such as looking into the mirror or adjusting their clothing. Research questions included: (1) Do scores on appearance monitoring, SPA, and concentration differ based on the style of athletic clothing worn (TR versus LC)?; (2) Do scores on clothing comfort differ based on the style of athletic clothing worn (TR versus LC)?; and (3) Are women’s athletic clothing comfort ratings associated with appearance monitoring, SPA, and concentration while controlling for clothing style, trait self-objectification, and body size perception?

For the first objective, we hypothesized that women wearing TR athletic clothing would engage in more appearance monitoring and report higher SPA and lower concentration than women wearing LC athletic clothing.^1,2,6,8,9,11^ For the second objective, despite the mixed findings in previous research,^12,16,17^ we hypothesized that women wearing TR clothing would report lower comfort ratings compared to women wearing LC clothing. Finally, for the third objective, we hypothesized that higher scores on clothing comfort (i.e., more comfortable) would be associated with lower scores on appearance monitoring and SPA and higher scores on concentration.^1,17–19^ Trait self-objectification, body size perception, and athletic clothing style were included as covariates in testing the third objective based on previously reported associations with the outcomes.^1,6,20,21^ Findings would have important implications for expanding our understanding of Self-Objectification Theory.^1^ Specifically, this study will provide novel experimental evidence on how contextual factors of clothing style and comfort elicit self-objectification and influence appearance monitoring, SPA, and concentration, offering insight into potential factors that may affect women’s cognitive and motor performance.

## Methods

This single-session, pseudorandomized, laboratory experimental investigation was part of a larger study examining athletic clothing style and body awareness on visual-motor performance and psychosocial and cognitive outcomes in women. The one set of outcomes (motor performance) and detailed methods have been previously documented^11^; none of the current outcomes (appearance monitoring, SPA, or concentration) have been previously reported. All study procedures were approved by University of Toronto Research Ethics Board (#00041818). Data collection occurred over a 10-month period spanning 2018–2019.

### Participants

Participants (*n* = 80) for the larger study were recruited through convenience sampling at a large university. Women were eligible to participate if they were between 18 and 35 years old, able to read and understand English, and had normal or corrected-to-normal vision. The analytical sample reported in the present paper consisted of 73 participants (7 participants from the original sample of 80 were not included in the analyses reported herein because they opted to exclude their video recording data used to assess appearance monitoring, a main outcome of the present study). The final sample in the present paper had an average age of 20.77 ± 3.15 years (range = 18–35) and average body mass index (BMI) of 22.73 ± 3.49 kg/m^2^ (range = 15.56–32.79). Most participants identified as single (93.2%, *n* = 68) and reported their highest level of education as high school diploma (42.5%, *n* = 31), some post-secondary (21.9%, *n* = 16), college diploma or certificate (1.4%, *n* = 1), undergraduate degree (27.4%, *n* = 20), or post-graduate degree (6.8%, *n* = 5). The sample identified as White (38.4%, *n* = 28), Chinese (26.0%, *n* = 19), South Asian (8.2%, *n* = 6), Black (6.8%, *n* = 5), Filipino (5.5%, *n* = 4), Arab (2.7%, *n* = 2), Korean (2.7%, *n* = 2), Latin American (1.4%, *n* = 1), Southeast Asian (1.4%, *n* = 1), West Asian (1.4%, *n* = 1), Japanese (1.4%, *n* = 1), and other cultural and racial background (5.5%, *n* = 4).

### Procedures

After written informed consent was obtained, participants were pseudorandomized to the TR or LC athletic clothing group. Random assignment was only constrained to ensure equal group sizes. Participants completed all measures during a single testing session in a lab at the university athletic centre, lasting approximately 60 min. Tasks were completed individually, guided by one of two young adult cisgender women experimenters. To mask the study purpose, women participants were told they were volunteering in a study on “people’s experiences wearing athletic clothing.” Participants completed a self-report survey containing demographic questions and measures assessing self-objectification constructs (e.g., trait self-objectification).^22^ Next, participants changed into one of two sets of athletic clothing in a changeroom. Clothing was either: (a) spandex shorts (7-inch rise, 5-inch inseam) and a spandex tank top (TR); or (b) polyester basketball shorts (12-inch rise, 5-inch inseam) and a cotton t-shirt (LC). Clothing items were black or dark blue, available in sizes XS to XL, and typical of clothing worn in athletic settings (see Supplemental Appendix B).^11^ Participants chose their clothing size within the assigned style.

Once wearing the clothing, body awareness was primed by the experimenter who took a digital photo of the participant and recorded their anthropometric measurements (i.e., height, weight, waist circumference). Participants then performed a computerized body size perception task^23^ followed by a visuomotor aiming task on a computer. Outcomes from the aiming task were collected for a specific purpose and are reported in another paper.^11^ For the present study, the aiming task provided participants opportunities to monitor their appearance while performing a task. Specifically, participants completed the aiming task while seated at a desk in a room with a full-length mirror positioned so their body remained visible in their peripheral visual field. Video cameras were visible to the participants, positioned on the top of the computer monitor and full-length mirror (see Supplemental Appendix C for a diagram of the experimental set-up). Participants completed 50 practice trials and two blocks of 50 experimental trials. After each main block of the aiming task, participants completed a manipulation check that assessed their attentional focus during the task. Following the computer tasks, participants answered a final survey that included measures of clothing comfort, SPA,^3,24^ and concentration.^25^ Participants received compensation ($10.00 CAD/h + ~ $2.50 CAD for performance on the aiming task), were debriefed on the study’s purpose, and offered the option to exclude any or all of their data.

### Measures

#### Demographics

Participants self-reported their age, highest level of education, relationship status, and ethnicity. Experimenter-measured height and weight were used to calculate BMI (kg/m^2^).

#### Covariates

##### Trait self-objectification

The Self-Objectification Questionnaire (SOQ)^22^ was used to measure trait self-objectification. The SOQ assesses whether a person views their body in an appearance-based (objectified) or competence-based (non-objectified) manner by asking participants to rank six appearance- (e.g., physical attractiveness) and six competence-based (e.g., physical fitness) attributes in order of importance to them on a scale of the most impact (rank = 1) to the least impact (rank = 12), with no ties permitted. Appearance and competence rankings were summed individually to produce an appearance score and a competence score. The final SOQ score was computed by subtracting the competence score from the appearance score to produce a score from −36 to 36. A higher score indicates greater trait self-objectification, reflecting a larger focus on appearance. Evidence of construct validity has been reported with young adults.^22^

##### Body size perception

Participants completed 10 trials of a computerized body size perception task to assess body size perception.^23^ They sat approximately 40 cm in front of a 22″ ViewSonic LCD touch-screen monitor (resolution: 1680 × 1050 pixels) and viewed a horizontally distorted version of an image of themselves wearing the assigned athletic clothing. To achieve distortion, digital images taken earlier were stretched or compressed in the horizontal dimension at a predetermined level ranging between 20% and 30% too wide or too thin (i.e., 70%, 80%, 120%, or 130% of the original image). Distortion level varied in each trial, creating 10 images with unique distortion amounts. Presentation order (level and direction of distortion) was maintained across participants. Using a two-button mouse, participants adjusted the image width (compress or stretch), whereby each button press changed the image by 1% per second. They were instructed to make as many adjustments as needed until they felt the image accurately represented their actual body size (i.e., 0% distortion or 100% original size). The average distortion present (assessed as percent distortion) after the participant adjusted the image width was computed. Higher scores reflect less accurate perceptions of actual body size.

#### Ratings of clothing comfort

Perceived comfort of the athletic clothing was assessed using the question: “How comfortable did the athletic clothing make you feel?” using a Likert-type scale of 1 = *very uncomfortable*, 2 = *slightly uncomfortable*, 3 = *neutral*, 4 = *slightly comfortable*, and 5 = *very comfortable*. Higher scores reflect greater feelings of clothing comfort.

#### Manipulation check

A study-specific item assessing attentional focus during the visuomotor aiming task was used as a manipulation check. Following each of the two blocks of the aiming task, participants responded to an item (“To what extent were you focused on your clothing”) on a Likert-type scale of 1 = *not at all* to 5 = *very much*. The items were averaged across blocks with higher scores reflecting a greater focus on the athletic clothing.

#### Main dependent variables

##### Appearance monitoring

Using the video recording, two raters independently counted and summed the number of instances in which participants overtly engaged in appearance monitoring using a researcher-created codebook. The codebook included seven coding features: gaze downward while sitting (i.e., looking at thighs/legs, chest, or stomach), gaze to one side of the body or the other while sitting (i.e., towards either arm), gaze into the mirror with eyes tracking in any direction, touch/rub/pinch any clothing, touch/rub/pinch any body part, touch/rub/pinch any facial area, and adjust hair, glasses, jewellery, or clothing. See Supplemental Appendix A for the codebook and seven steps used to code for appearance monitoring. The raters coded and counted all appearance monitoring behaviours throughout all five sections of the experiment (computerized body size perception task, aiming task block 1, manipulation check 1, aiming task block 2, and manipulation check 2), and then summed these counts to generate a single value.

A pilot test of the first five videos was completed independently by raters, and a meeting was held with the first author to ensure consistent interpretations of coding features. No changes were made to the codebook, and raters completed coding for the remaining videos. Interrater reliability was computed using a two-way mixed, absolute, average-measures intraclass correlation coefficient (ICC)^26,27^ to assess the degree that the two raters were consistent in their frequency counts of appearance monitoring across subjects. To calculate the ICCs, a sum of frequencies across the five time points was calculated for each coding feature and a separate ICC was calculated per coding feature (seven ICCs). The resulting ICCs ranged from 0.87 to 1.00 (ICCs: gaze down = 1.00, gaze side = 0.95, gaze mirror = 1.00, touch clothing = 0.87, touch body = 0.98, touch face = 0.91, adjust = 0.98). Following ICC calculation, the first author resolved discrepancies by reviewing relevant video recordings. No discrepancies remained following this review. Scores were summed across the seven codes to yield a total score wherein higher scores represent more appearance monitoring.

##### Social physique anxiety

Participants completed the abbreviated four-item version^24^ of the Social Physique Anxiety Scale (SPAS).^3^ The SPAS measures the degree to which a person experiences anxiety regarding their physique in social situations. Participants rated the extent to which they were experiencing the feelings described by each item (e.g., “I am comfortable with the appearance of my physique/figure”) on a 5-point Likert-type scale ranging from 1 = *not at all* to 5 = *extremely*. Items were reverse-scored and summed so that a higher score reflects greater SPA. Evidence of internal consistency was previously reported.^24^ In the present study, internal consistency was ω = 0.88.

##### Concentration

Concentration was assessed using the concentration on task at hand subscale of the Flow State Scale.^25^ Participants were asked to report the extent to which they agreed with each of the four statements as related to their experience in the aiming task (e.g., “My attention was focused entirely on what I was doing”) on a Likert-type scale from 1 = *strongly disagree* to 5 = *strongly agree*. The four items were summed with higher scores representing higher concentration. Evidence of internal consistency has been demonstrated among adolescents and adults.^25^ In the current study, internal consistency was ω = 0.96.

### Data analysis

Analyses were conducted in IBM Statistical Package for the Social Sciences (version 28). Data were screened for missing data, normality, and outliers. Descriptive statistics (frequencies, means, standard deviations) were calculated to characterize the sample. Pearson’s bivariate correlations and reliability coefficients (ω)^28^ of appropriate scales were computed. A Mann-Whitney *U* test was calculated to assess whether scores on the manipulation check differed between clothing style groups (TR versus LC). To address objective 1 (i.e., examine group differences in self-objectification constructs between athletic clothing styles), independent samples *t*-tests were conducted to test whether appearance monitoring, SPA, and concentration differed between clothing style groups (TR versus LC). Welch’s *t*-tests were used to account for unequal variances between groups. Cohen’s *d* effect sizes were computed and interpreted using accepted guidelines (i.e., small = 0.20, medium = 0.50, and large = 0.80).^29^ To address objective 2 (i.e., examine group differences in clothing comfort between athletic clothing styles), an independent samples *t*-test was computed to assess whether clothing comfort ratings differed between clothing style groups (TR versus LC). To address objective 3 (i.e., examine the association between clothing comfort ratings and self-objectification constructs), three stepwise regressions were performed to test the association between clothing comfort and appearance monitoring, SPA, and concentration separately. The covariates of clothing style, trait self-objectification, and body size perception were added in step 1, clothing comfort was added in step 2, and appearance monitoring, SPA, or concentration (depending on the outcome of the model) was included as the dependent variable. An a-priori power analysis indicated that a minimum sample size of 55 would provide sufficient power (0.80) to detect a medium effect (Cohen’s *f*^2^ = 0.15) with an alpha level of *p* = 0.05.^30^ A medium effect size was selected to balance theoretical plausibility and feasible recruitment.^29^ The STROBE checklist was referenced for guidance on reporting study findings.^31^

## Results

Data from 73 participants comprised the final analytic sample and were screened for missing data. One score was missing for BMI and was addressed using mean imputation. No other missing data were identified. Four univariate outliers (i.e., values greater than ±3 standard deviations) were detected on body size perception (two outliers) and appearance monitoring (two outliers) and associated participant scores were winsorized.^32^

Among the 73 participants, 37 (50.7%) wore LC athletic clothing and 36 (49.3%) wore TR athletic clothing. Participants in the TR group reported significantly higher scores on the manipulation check relative to the LC group (*U* = 457.50, *Z* = −2.51, *p* = 0.012, *r* = 0.29), indicating the TR group had a greater focus on their body during the visuomotor aiming task than the LC group. Descriptive statistics and bivariate correlations for the main study variables are presented in Table 1. Notably, clothing comfort was positively correlated with concentration (*r* = 0.35, *p* = 0.003) and negatively correlated with SPA (*r* = −0.31, *p* = 0.008).

**Table 1.** Descriptive statistics and bivariate correlations [95% confidence intervals] (*n* = 73).

Variables	1	2	3	4	5	6	7	8
1. Clothing comfort	—							
2. Appearance monitoring	−0.06 [−0.28, 0.17]	—						
3. Concentration	0.35** [0.13, 0.54]	0.05 [−0.19, 0.27]	—					
4. Social physique anxiety	−0.31** [−0.50, −0.08]	−0.02 [−0.25, 0.21]	−0.22 [−0.43, 0.01]	—				
5. Body size perception	−0.003 [−0.23, 0.23]	0.07 [−0.16, 0.30]	0.03 [−0.20, 0.26]	0.27*[0.05, 0.47]	—			
6. Trait self-objectification	−0.06 [−0.29, 0.17]	−0.04 [−0.27, 0.19]	−0.01 [−0.24, 0.22]	0.33** [0.11, 0.52]	0.19 [−0.04, 0.41]	—		
7. Clothing style^a^	0.27* [0.03, 0.47]	−0.21 [−.042, 0.03]	−0.08 [−0.31, 0.16]	0.08 [−0.16, 0.31]	−0.12 [−0.34, 0.13]	0.21 [−0.03, 0.43]	—	
8. Manipulation check^b^	−0.44** [−0.61, −0.24]	0.23* [0.003, 0.44]	−0.29* [−0.49, −0.07]	−0.05 [−0.27, 0.19]	−0.02 [−0.25, 0.22]	−0.15 [−0.37, 0.08]	−0.30* [−0.50, −0.06]	—
Mean	3.73	7.30	15.53	12.89	−10.51	−5.67	—	1.55
Standard deviation	1.18	7.92	4.49	3.42	5.34	14.30	—	0.78
Score range	1 to 5	0 to 32	5 to 20	5 to 20	−29.37 to 8.19	−29 to 30	—	1 to 4

a Spearman rho correlation coefficients; coded as 1 = tight and revealing; 2 = loose and concealing.

b Participants answered “To what extent were you focused on your clothing” from 1 = *not at all* to 5 = *very much*.

* *p* < 0.05. **p < 0.01.

### Main results

#### Objective 1: clothing style on appearance monitoring, SPA, and concentration

Means, standard deviations, and effect sizes for appearance monitoring, SPA, and concentration between athletic clothing style groups are displayed in Table 2. Based on the results of the independent samples *t*-test, participants wearing TR clothing engaged in significantly more appearance monitoring relative to those wearing LC clothing (*M*_TR_ = 9.30, SD = 9.23; *M*_LC_ = 5.35, SD = 5.88; *t*(59.1) = 2.17, *p* = 0.034, *d* = 0.51). There were no significant clothing group differences for SPA (*d* = 0.16) or concentration (*d* = 0.25).

**Table 2.** Means, standard deviations, and Cohen’s *d* [95% confidence intervals] of variables by clothing style.

Variables	Clothing style		
	Tight and revealing (*n* = 36)	Loose and concealing (*n* = 37)	*d* [95% CI]
	*M* ± SD		
Appearance monitoring	9.30 ± 9.23	5.35 ± 5.88	**0.51 [0.04, 0.98]**
Social physique anxiety	12.61 ± 3.63	13.16 ± 3.24	0.16 [−0.62, 0.30]
Concentration	16.11 ± 3.85	14.97 ± 5.02	0.25 [−0.21, 0.71]
Clothing comfort	3.42 ± 1.18	4.03 ± 1.12	**0.53 [−1.00, −0.06]**

Bolded numbers indicate values that statistically differed between groups (*p* < 0.05). CI: confidence interval; SD: standard deviation.

#### Objective 2: clothing style on clothing comfort

Means, standard deviations, and effect sizes for clothing comfort between athletic clothing style groups are presented in Table 2. Based on the independent samples *t*-test results, women wearing TR clothing reported significantly lower scores on clothing comfort relative to those wearing LC clothing (*M*_TR_ = 3.42, SD = 1.18; *M*_LC_ = 4.03, SD = 1.12; *t*(71) = −2.27, *p* = 0.026, *d* = 0.53).

#### Objective 3: clothing comfort and appearance monitoring, SPA, and concentration

The overall models examining the associations between clothing comfort and SPA (*R*^2^ = 0.25, *F*(4, 68) = 5.75, *p* < 0.001) and clothing comfort and concentration (*R*^2^ = 0.18, *F*(4, 68) = 3.63, *p* = 0.010) were significant, accounting for 25% and 18% of the variance in SPA and concentration, respectively. After controlling for body size perception, trait self-objectification, and clothing style, higher clothing comfort ratings (i.e., more comfortable) predicted lower SPA (β = −0.33, *p* = 0.004) and higher concentration (β = 0.42, *p* < 0.001). The semi-partial correlations for clothing comfort were sr = −0.31 (sr^2^ = 0.10) for SPA and sr = 0.40 (sr^2^ = 0.16) for concentration, reflecting approximately 10% and 16% of the unique variance, respectively. Clothing comfort ratings did not significantly predict appearance monitoring. Table 3 presents all stepwise regression results.

**Table 3.** Stepwise regression analyses predicting appearance monitoring, social physique anxiety, and concentration.

Variables	Model information	*B* (SE)	β	*t* [95% CI]
Appearance monitoring				
Step 1	*F*(3, 69) = 1.63, *p* = 0.190, *R*^2^ = 6.6%			
Body size perception		0.08 (0.18)	0.06	0.48 [−0.27, 0.44]
Trait self-objectification		−0.00 (0.07)	−0.01	−0.06 [−0.14, 0.13]
Clothing style		−3.86 (1.88)	−0.25	−2.06* [−7.61, −0.12]
Step 2	*F*(4, 68) = 1.21, *p* = 0.317, Δ*F* = 0.00, *R*^2^ = 6.6%			
Body size perception		0.08 (0.18)	0.06	0.47 [−0.27, 0.44]
Trait self-objectification		−0.00 (0.07)	−0.01	−0.06 [−0.14, 0.13]
Clothing style		−3.87 (1.97)	−0.25	−1.96 [−7.81, 0.07]
Clothing comfort		0.01 (0.82)	0.00	0.01 [−1.63, 1.65]
Social physique anxiety				
Step 1	*F*(3, 69) = 4.20, *p* = 0.009, *R*^2^ = 15.4%			
Body size perception		0.14 (0.07)	0.22	1.96 [−0.003, 0.29]
Trait self-objectification		0.07 (0.03)	0.28	2.39* [0.01, 0.12]
Clothing style		0.29 (0.77)	0.04	0.38 [−1.25, 1.84]
Step 2	*F*(4, 68) = 5.75, *p* < 0.001, Δ*F* = 8.95, *R*^2^ = 25.3%			
Body size perception		0.15 (0.07)	0.24	2.19* [0.01, 0.29]
Trait self-objectification		0.06 (0.03)	0.24	2.13* [0.004, 0.11]
Clothing style		0.93 (0.76)	0.14	1.23 [−0.59, 2.46]
Clothing comfort		−0.95 (0.32)	−0.33	−2.99** [−1.58, −0.32]
Concentration				
Step 1	*F*(3, 69) = 0.40, *p* = 0.754, *R*^2^ = 1.7%			
Body size perception		0.02 (0.10)	0.02	0.18 [−0.19, 0.22]
Trait self-objectification		0.00 (0.04)	0.01	0.11 [−0.07, 0.08]
Clothing style		−1.15 (1.09)	−0.13	−1.05 [−3.33, 1.03]
Step 2	*F*(4, 68) = 3.63, *p* = 0.010, Δ*F* = 13.11, *R*^2^ = 17.6%			
Body size perception		0.00 (0.10)	0.00	0.04 [−0.19, 0.19]
Trait self-objectification		0.02 (0.04)	0.07	0.57 [−0.05, 0.09]
Clothing style		−2.22 (1.05)	−0.25	−2.11* [−4.31, −0.12]
Clothing comfort		1.58 (0.44)	0.42	3.62** [0.71, 2.45]

CI: confidence interval; SE: standard error.

* *p* < 0.05. **p < 0.01.

## Discussion

Drawing from Objectification Theory,^1^ the data reported in the present paper were analysed to examine the differences in athletic clothing style (TR versus LC) on appearance monitoring, SPA, concentration, and clothing comfort among women. We also tested the association between clothing comfort and appearance monitoring, SPA, and concentration. Aligned with our hypothesis, Objectification Theory,^1^ and prior literature,^2,9,11^ women who wore TR athletic clothing engaged in significantly more appearance monitoring relative to those who wore LC clothing. This result extends findings from previous experimental studies^8^ by demonstrating a link between athletic clothing and actual appearance monitoring. Given that appearance monitoring may divert the cognitive resources needed for a task,^2,9,11^ future research is needed to explore the impact of objective appearance monitoring, as a behavioural expression of state self-objectification, on cognitive and motor task performance outcomes. To our knowledge, this is the first study to develop and apply a behavioural method for assessing appearance monitoring, offering a unique approach to understanding these relationships.

Contrary to our hypotheses, SPA and concentration did not differ between clothing styles. Dimas et al.^6^ reported findings that were different from the present study in that they reported a significant difference between clothing style, and SPA and intrinsic motivation for balance tasks (used as an indicator of flow). The clothing manipulation in the previous study, however, involved a swimsuit (TR) versus a sweater (LC), whereas athletic clothing was used in the present study. It may be that the two athletic clothing styles in the present study were not different enough from each other to significantly affect SPA and concentration. Researchers could replicate the present study using more revealing and less revealing athletic clothing (e.g., sports bra and spandex shorts versus loose long-sleeve top and track pants). Furthermore, given the relevance of athletic clothing in sport contexts, it may also be valuable to examine the differences between mandated sport-specific uniforms (e.g., swimming/volleyball uniforms versus basketball/soccer uniforms) on appearance monitoring, SPA, and concentration.

Aligned with hypotheses and previous studies,^12,16^ women wearing TR clothing reported significantly lower scores on clothing comfort relative to those wearing LC clothing, indicating a preference for LC clothing for comfort. Despite the significant differences, frequencies demonstrate that participants’ comfort perceptions do not neatly align with the clothing style worn. For example, while most (76%) participants wearing LC clothing rated the athletic clothing as comfortable or very comfortable, some (14%) found LC athletic clothing uncomfortable or very uncomfortable. These nuances support previous qualitative work wherein women’s accounts highlight individual differences in their perceptions of clothing styles.^16,17^ Perceptions of clothing may also be influenced by broader sociocultural influences, as activewear marketing and fitness media frequently depict women wearing TR clothing, often emphasizing thin and toned body ideals and appearance-focused evaluations of the body.^33–35^ Overall, this result has implications for experimental studies that manipulate clothing style to induce state self-objectification among women. While the level of comfort may be consistent across clothing styles for *most* women, individual differences in clothing comfort are experienced, which suggests that researchers should consider measuring comfort ratings when experimentally manipulating clothing style.

In fact, it may be women’s perceptions of the athletic clothing, rather than the clothing itself, that predict SPA and concentration. In line with our hypotheses, lower clothing comfort ratings (more uncomfortable) were related to higher SPA and lower concentration. Previous literature suggests that clothing discomfort is associated with a reduction in cognitive resources necessary to complete tasks.^18,19^ Thus, it is plausible that wearing uncomfortable clothing increases body awareness, which may promote anxiety related to perceived negative appearance evaluations.^4^ This heightened SPA could then have negative implications for cognitive performance.^7^ Regarding concentration, our finding mirrors qualitative accounts wherein recreationally active women said comfortable clothing helped them focus on their activity rather than their appearance.^17^ Indeed, previous correlational research demonstrated that women who prioritized clothing comfort reported lower self-objectification; possibly because they focused more on how their body felt rather than how it looked.^36^ In the current study, participants who reported greater comfort were able to dedicate more cognitive resources to the visuomotor performance task, likely leading to a greater focus on their body’s function. Taken together, these findings contribute to our understanding of Objectification Theory by highlighting the importance of clothing comfort perceptions for women.

Although perceived athletic clothing comfort was significantly associated with SPA and concentration, it was not related to participants’ engagement in appearance monitoring. This finding is surprising given that appearance monitoring is a core facet of self-objectification.^1^ Recall, however, that there was a difference between the TR and LC clothing groups on appearance monitoring behaviours. It could be that the measure of appearance monitoring used (i.e., overt behaviours of gaze, touch, and adjustments) did not capture all potential ways participants focused on their appearance, perhaps reflecting clothing style rather than comfort. Nonetheless, participants may have engaged in covert forms of appearance monitoring (e.g., thinking about one’s appearance)^37^ that were not captured by measuring overt behaviour. Indeed, comfort ratings were negatively correlated with the manipulation check item (i.e., “To what extent were you focused on your clothing”) suggesting that those who rated the clothing as more uncomfortable also reported focusing on it more. This finding supports the contention that covert forms of appearance monitoring should also be assessed when examining clothing preferences. In addition, while the objective assessment of appearance monitoring was novel and a strength of this study, aspects of the procedure may be altered in future work. For instance, the consent form stated that video recording would occur (though participants were not reminded until debrief), and cameras were visible, with some participants looking directly into them, suggesting awareness that may have impacted overt appearance monitoring. Furthermore, participants were alone in the testing room for most of the experiment. Based on experimenter observations, many participants engaged in more appearance monitoring (e.g., mirror gazing, adjusted clothing) right before opening the testing room door to notify the experimenter that they had completed a step in the protocol. Potential evaluation from another person may have increased appearance monitoring behaviours,^1^ suggesting it would be worthwhile to compare appearance monitoring when others are versus are not present. Overall, further research is needed to clarify the relationship between appearance monitoring and athletic clothing comfort.

### Study limitations

There are limitations to the study that are worth noting. Participants were young adult women who mainly identified as White or Chinese and mostly fell within the ‘healthy weight’ range based on BMI. Thus, our results are difficult to generalize to other gender identities, ethnicities, or individuals outside the ‘healthy weight’ BMI range. Researchers are encouraged to examine these relationships among more diverse samples. In addition, self-identified gender was not collected. Self-objectification differs across gender identities;^10,38^ therefore, more research across the gender spectrum is needed. While the young adult age range is a key developmental phase where experiences of sexual objectification are heightened and vulnerability to self-objectification increases,^39^ these relationships should also be explored among other age groups (e.g., adolescence) to understand these phenomena across ages. Finally, based on the clothing provided, the features of TR and LC cannot be teased apart. It may be worthwhile to examine varying combinations of athletic clothing as prior literature has demonstrated differences in self-objectification for clothing that is tight and concealing and loose and revealing compared to TR and LC.^12^

### Implications and future directions

The novel findings in this study extend tenets of Objectification Theory^1^ by identifying athletic clothing discomfort as a potential elicitor of self-objectification in women and athletic clothing style and comfort as predictors of appearance monitoring, SPA, and concentration. These results further suggest that researchers should consider individual differences in women’s perceptions of athletic clothing, such as clothing comfort, as these differences are associated with self-objectification consequences (i.e., higher SPA, lower concentration). In addition to clothing comfort, other perceptions of athletic clothing, including functionality, could be examined in future studies. These results also build on prior qualitative research demonstrating that adolescent girls and women reported sport uniforms as impacting their body awareness, body image, and were cited as harmful for their performance and a deterrent for continuing sport participation.^40,41^ As such, appearance monitoring, SPA, and concentration may influence cognitive and motor performance.^1,6,7^ Researchers should continue to examine the extent to which athletic clothing style and comfort are associated with women’s body image, cognitive resources, and cognitive and motor performance.

Future research should also consider how broader sociocultural influences, such as activewear marketing and media imagery, shape women’s perceptions of athletic clothing. Activewear advertising and fitness-related social media content frequently portray idealized bodies in TR clothing.^33–35,42^ Extending this line of research, a recent study using artificial intelligence (AI)-generated images of athlete and non-athlete bodies found that athlete images reinforced ideal body standards of thinness and muscularity and depicted more revealing clothing than non-athlete images.^43^ Experimental evidence has demonstrated that exposure to activewear retail imagery can increase a focus on appearance and negatively influence women’s body image.^34,35^ Research on social media fitness imagery further indicates that viewing fitspiration content can increase appearance comparison and body dissatisfaction among women.^44^ Within this broader sociocultural context, the normalization of TR athletic clothing, potentially amplified by AI-generated imagery, may shape women’s perceptions and influence clothing comfort ratings and appearance monitoring. Investigating how the consumption of activewear imagery interacts with clothing style and comfort to influence appearance monitoring, SPA, and concentration could provide additional context to the relationships between athletic clothing and women’s body image.

Furthermore, these findings may have practical implications for exercise and sport participation. When athletic clothing was TR or considered more uncomfortable, participants focused more on their appearance, experienced higher SPA, and reported lower concentration on the aiming task. Thus, women wearing exercise clothing or sport uniforms that are TR or perceived as more uncomfortable may reallocate cognitive resources, leading to injury or diminished performance. As discussed in Cox et al.,^11^ women who allocate greater cognitive resources towards the body’s appearance relative to athletic performance may experience negative performance outcomes that demotivate further athletic engagement and learning long-term. Therefore, developing and implementing strategies to reduce the impact of clothing on self-objectification, appearance monitoring, and related consequences is needed. Involving women in sport uniform design and encouraging women to wear athletic clothing they find comfortable can improve clothing comfort perceptions, reducing the potential for negative consequences.^40^ In addition, women who have lower autonomy in athletic clothing choices (e.g., sport uniforms) may benefit from mindfulness, breathing techniques, or yoga, which show promise for reducing self-objectification and appearance monitoring in women.^45,46^ Women, as well as coaches, personal trainers, and group fitness instructors, are encouraged to implement these individual-level strategies in an attempt to disrupt the negative influence of athletic clothing on women’s body image and athletic performance.

Finally, this manuscript is the first known study to report a method for assessing behavioural indices of appearance monitoring. As outlined in Supplemental Appendix A, we developed and implemented a method in which videos of participants were coded for actions linked to appearance monitoring including clothing adjustments, mirror checks, and gazes at the self. This systematic video analysis could be adopted by researchers to gain new and comprehensive insights into the complex characteristics of clothing and environments that may increase appearance monitoring. In addition, this approach may be used to provide a more nuanced understanding to the relationships between appearance monitoring and other psychological constructs (e.g., appearance emotions, affect/mood) that were beyond the scope of the present study.

## Conclusion

The current study was designed to manipulate the athletic clothing worn by women and assess the influence of clothing style on appearance monitoring, SPA, concentration, and ratings of clothing comfort. In addition, the relationships between clothing comfort and appearance monitoring, SPA, and concentration were examined. Overall, these results extend Objectification Theory^1^ by providing evidence for the importance of athletic clothing comfort and suggesting clothing style and comfort may influence women to focus more on their appearance, experience higher SPA, and concentrate less on aiming tasks. Further, by introducing a novel method to assess appearance monitoring, this study: (1) provides researchers with an approach to objectively assess appearance monitoring; and (2) generates experimental evidence relating women’s experiences of wearing athletic clothing with a behavioural expression of state self-objectification. Together, these findings highlight the importance of developing and implementing strategies that reduce the potential for athletic clothing to elicit self-objectification and related consequences for women. Addressing clothing comfort and design may represent a practical avenue for supporting women’s body image and its potential cognitive and performance-related consequences.

## Supplemental Material

sj-docx-1-whe-10.1177_17455057261443139 – Supplemental material for Athletic clothing style and comfort: Associations with appearance monitoring, social physique anxiety, and task concentration among women Supplemental material, sj-docx-1-whe-10.1177_17455057261443139 for Athletic clothing style and comfort: Associations with appearance monitoring, social physique anxiety, and task concentration among women by Madison F. Vani, Catherine M. Sabiston, Elizabeth Cox, April Karlinsky and Timothy N. Welsh in Women's Health

sj-docx-2-whe-10.1177_17455057261443139 – Supplemental material for Athletic clothing style and comfort: Associations with appearance monitoring, social physique anxiety, and task concentration among women Supplemental material, sj-docx-2-whe-10.1177_17455057261443139 for Athletic clothing style and comfort: Associations with appearance monitoring, social physique anxiety, and task concentration among women by Madison F. Vani, Catherine M. Sabiston, Elizabeth Cox, April Karlinsky and Timothy N. Welsh in Women's Health

sj-docx-3-whe-10.1177_17455057261443139 – Supplemental material for Athletic clothing style and comfort: Associations with appearance monitoring, social physique anxiety, and task concentration among women Supplemental material, sj-docx-3-whe-10.1177_17455057261443139 for Athletic clothing style and comfort: Associations with appearance monitoring, social physique anxiety, and task concentration among women by Madison F. Vani, Catherine M. Sabiston, Elizabeth Cox, April Karlinsky and Timothy N. Welsh in Women's Health

sj-docx-4-whe-10.1177_17455057261443139 – Supplemental material for Athletic clothing style and comfort: Associations with appearance monitoring, social physique anxiety, and task concentration among women Supplemental material, sj-docx-4-whe-10.1177_17455057261443139 for Athletic clothing style and comfort: Associations with appearance monitoring, social physique anxiety, and task concentration among women by Madison F. Vani, Catherine M. Sabiston, Elizabeth Cox, April Karlinsky and Timothy N. Welsh in Women's Health
