# Supplementary material for: Athletic clothing style and comfort: Associations with appearance monitoring, social physique anxiety, and task concentration among women
Source: Womens Health (Lond). 2026 Apr 18;22:17455057261443139. doi: 10.1177/17455057261443139 (PMC13100385; doi:10.1177/17455057261443139)
Supplement: sj-docx-1-whe-10.1177_17455057261443139 – Supplemental material for Athletic clothing style and comfort: Associations with appearance monitoring, social physique anxiety, and task concentration among women [file sj-docx-1-whe-10.1177_17455057261443139.docx]

**Appendix A. Appearance Monitoring Codebook and Instructions.**

The following seven-step codebook was used to code for Appearance Monitoring in the present study. For use of this codebook, please cite [final reference here]. Report any modifications made to the original codebook in the Methods section.

**Step 1.** **Define Coding Features**

Create a codebook with coding features, definitions, and notes. See below for the Coding Features and Definitions of the present study:

| **Coding Theme** | **Coding Feature** | **Definition** | **Notes** |
| --- | --- | --- | --- |
| Gaze | Gaze Down | Gaze downward while sitting (looking at thighs/legs, chest, or stomach) | For Gaze, count as one regardless of length of gaze until they redirect their gaze elsewhere. |
|  | Gaze Side | Gaze to one side or the other while sitting (towards either arm) |  |
|  | Gaze Mirror | Gaze into mirror with eyes tracking in any direction |  |
| Touch/  Adjust | Touch Clothing | Touch/rub/pinch any clothing | For Touch/Adjust, a touch/rub/pinch/adjust for any length of time is counted as 1 until the location of touch/adjust changes or there is a lag of time of at least 1 second:   - A change of location can be understood as when the touch/rub/pinch/adjust moves at least 1 inch from the originating location. - A lag time can be understood as when the person moves their hand from a location and then returns to it at least 1 second later.   If it is difficult to discern touch vs. adjust clothing, code as Touch Clothing unless it is an obvious adjustment of the clothing. |
|  | Touch Body Part | Touch/rub/pinch any body part |  |
|  | Touch Face | Touch/rub/pinch any facial area |  |
|  | Adjust | Adjust hair, glasses, jewellery, clothing |  |

Outline the features that will not be coded. For our study, we did not code for unintentional body checking behaviours including itching on body/face/head, brushing hair out of face, rubbing eyes, gaze at camera, shifting of position or movement, and resting hand on face.

**Step 2. Confirm Timeframes for Coding**

Decide if you are breaking up coding based on the timeframe in the study. For example, in the present study, we had 5 timeframes: during the computerized body size perception task, aiming task block 1, manipulation check 1, aiming task block 2, and manipulation check 2.

**Step 3.** **Create Spreadsheet**

Create a Spreadsheet that will be used to code appearance monitoring. A simplified example of the Spreadsheet used for the present study is provided below:

| Participant ID | Gaze Down | Gaze Side | Gaze Mirror | Touch Clothing | Touch Body Part | Touch Face | Adjust | Total | Notes |
| --- | --- | --- | --- | --- | --- | --- | --- | --- | --- |
|  |  |  |  |  |  |  |  |  |  |

*Note.* If there are separate time points in the experiment to code for, have a separate column for each time point x coding feature.

**Step 4. Pilot the Coding Features**

Two raters should pilot test the first five videos independently using the Spreadsheet and Coding Features definitions.

**Step 5. Pilot Meeting**

Hold a meeting with the two raters and a third team member to discuss any issues from piloting. Discuss whether changes to the codebook are necessary and discuss discrepancies among the team to ensure interpretation of all coding features are consistent across raters.

**Step 6. Complete Coding**

After the pilot meeting, the two raters should independently complete coding for the remaining videos.

**Step 7. Final Meeting**

Hold a meeting with the two raters and a third team member to discuss any discrepancies. Resolve discrepancies through discussion, or if needed, by a decision from the third team member. Retain original codebook sheets to calculate appropriate inter-rater reliability between the two raters.
