# Supplementary material for: Athletic clothing style and comfort: Associations with appearance monitoring, social physique anxiety, and task concentration among women
Source: Womens Health (Lond). 2026 Apr 18;22:17455057261443139. doi: 10.1177/17455057261443139 (PMC13100385; doi:10.1177/17455057261443139)
Supplement: sj-docx-2-whe-10.1177_17455057261443139 – Supplemental material for Athletic clothing style and comfort: Associations with appearance monitoring, social physique anxiety, and task concentration among women [file sj-docx-2-whe-10.1177_17455057261443139.docx]

**Appendix B.** **Athletic Clothing Worn by Participants.**


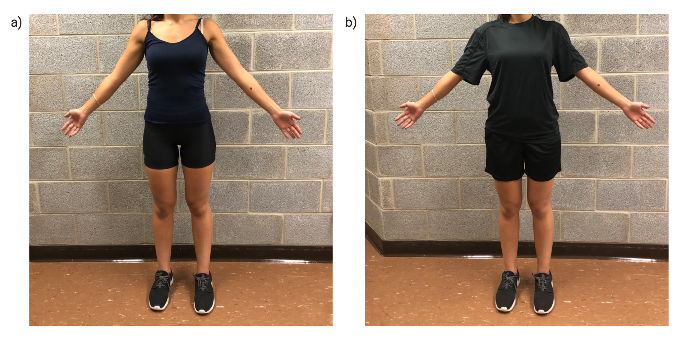


*Note.* Participants wore (a) tight/revealing or (b) loose/concealing athletic clothing by random assignment.
