# Supplementary material for: Athletic clothing style and comfort: Associations with appearance monitoring, social physique anxiety, and task concentration among women
Source: Womens Health (Lond). 2026 Apr 18;22:17455057261443139. doi: 10.1177/17455057261443139 (PMC13100385; doi:10.1177/17455057261443139)
Supplement: sj-docx-3-whe-10.1177_17455057261443139 – Supplemental material for Athletic clothing style and comfort: Associations with appearance monitoring, social physique anxiety, and task concentration among women [file sj-docx-3-whe-10.1177_17455057261443139.docx]

**Appendix C.** **Schematic Diagram of Experimental Set-up.**


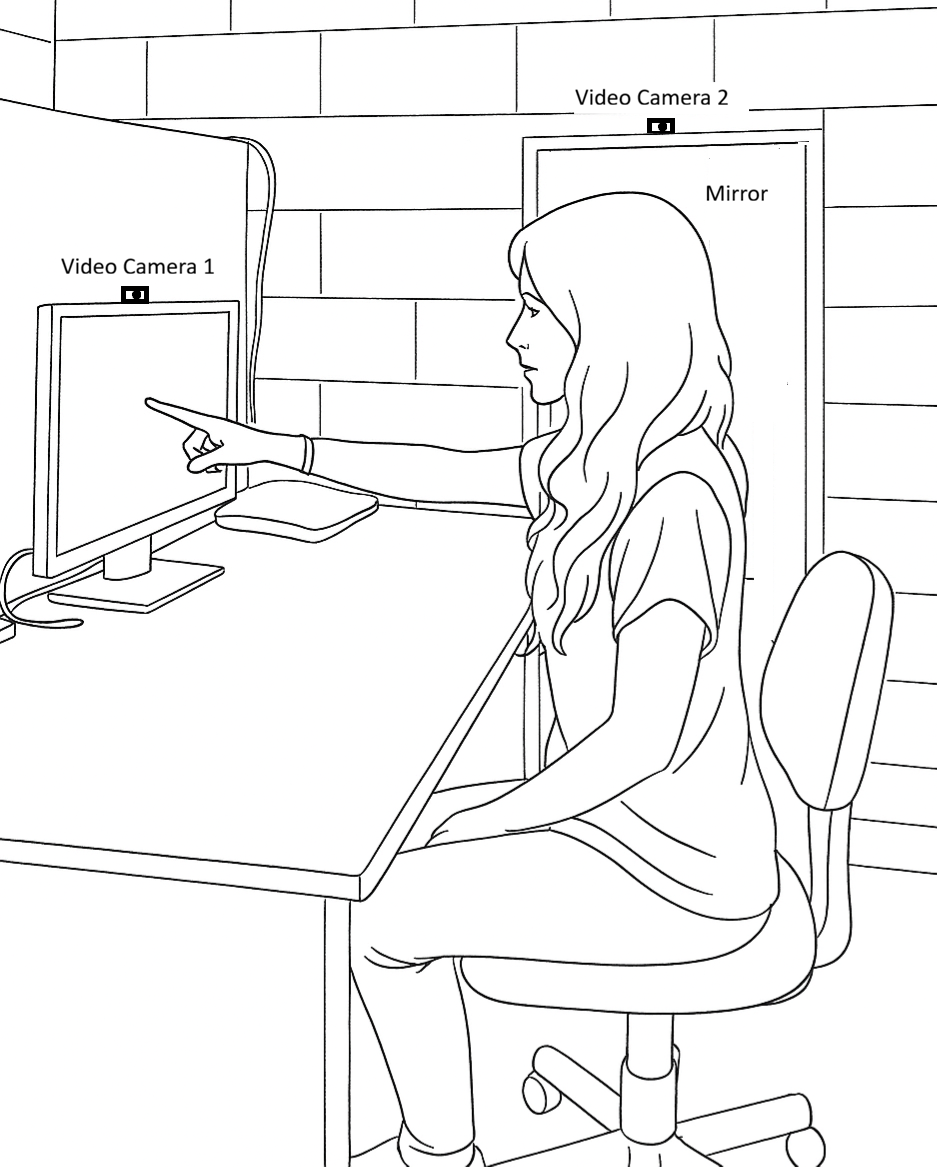


*Note.* Artificial Intelligence (i.e., Microsoft Co-Pilot) was used to convert a real image to a line drawing for clarity and to protect anonymity.

.
